# Effect of bupivacaine soaked gauze in postoperative pain relief in laparoscopic cholecystectomy: a prospective observational controlled trial in 120 patients

**DOI:** 10.1186/s13037-015-0077-2

**Published:** 2015-09-15

**Authors:** Aman Ahmad, Salman Faridi, Faisal Siddiqui, Muhammad Muzzammil Edhi, Mehmood Khan

**Affiliations:** Department of General surgery, Liaquat national hospital and medical college, Karachi, Pakistan; Intern, Liaquat national hospital and medical college, Karachi, Pakistan; Intern, Dhaka medical college, Karachi, Pakistan

## Abstract

**Background:**

Symptomatic gallstone disease is one of the most common problem attended by a general surgeon. The application of minimally invasive surgical techniques for the removal of gallbladder is now an accepted and preferred method for treating this condition. The avoidance of a subcostal incision and minimal bowel handling leads to decreased postoperative pain, early returning to function and overall shorter duration of hospital stay. Nevertheless, patients do have significant postoperative pain, and newer techniques to further reduce this pain are the subject of many ongoing studies. Many intraoperative techniques for reducing postoperative pain have been described. The current practice at many institutions, including ours, is to discharge the patient on the first postoperative day on oral analgesics. Better control of postoperative pain may help establishing laparoscopic cholecystectomy as a day care procedure in selected patients. The aim of this study is to determine the effect of 0.5 % bupivacaine soaked oxidized regenerated cellulose surgical versus normal saline soaked surgical applied at the gallbladder bed on postoperative mean pain score after laparoscopic cholecystectomy for symptomatic gallstones.

**Patients and methods:**

Patients scheduled for laparoscopic cholecystectomy were enrolled in the study after meeting the inclusion criteria. Relevant history was taken and clinical examination was done. Necessary investigations were carried out. All patients were divided to receive either 0.5 % bupivacaine soaked surgicel or normal saline soaked surgicel after laparoscopic cholecystectomy with each group having equal number of patients. The pain score was measured with a visual analogue scale (VAS) at 4, 12 and 24 h after the procedure in both groups. All data was recorded on performa and SPSS-19 was used for analysis.

**Results:**

The demographic characteristic of the two groups has shown that studied patients were matched as regarding gender, age, weight, ASA status and duration of surgery. Post-operative abdominal pain was significantly lower in **bupivacaine** Group than Saline group. This difference was reported from 4 h till 12 h post-operatively. Bradycardia, Hypotension and Urinary retention were the most common perioperative symptoms reported, with an incidence of 28.3 % in the saline Group and 15 % in the **bupivacaine** group with no significant differences. Evaluation of postoperative details such as oral intake, time to walk and length of hospital stay revealed that bupivacaine group reported better outcomes as compared to saline group.

**Conclusion:**

Placing bupivacaine soaked surgicel has been shown to decrease the mean postoperative pain score in patients. No significant complication was noticed with the use of surgicel. Because adequate pain control requires intravenous medications, additional methods for pain control need to be studied before laparoscopic cholecystectomy can be routinely performed as a day care case.

## Introduction

Although laparoscopic cholecystectomy has numerous benefits over the open method, still postoperative pain remains an issue. Postoperative pain, requiring injectable analgesics prolong the patient’s hospital stay, and is one of the hurdles in performing laparoscopic cholecystectomy as a day case. The pain reaches a maximum level within 6 h of the procedure and then gradually decreases over a couple of days, but varies considerably between patients [1, 2]. Studies show that postoperative analgesics may be required in 58 % to as much as in more than 70 % of patients [3–5].

Many methods have been tried to decrease the postoperative pain, like low-pressure pneumoperitoneum, gasless technique, local anesthetic infiltration, saline washout and instillation of a local anesthetic agent in the subdiaphragmatic area [6–8].

The pain that a patient feels after laparoscopic cholecystectomy can be divided into 3 types: Visceral, parietal and shoulder pain which have different intensities [9]. The visceral pain is because of the surgical dissection and tissue handling at the gall bladder bed. The parietal or somatic pain is caused by the trauma to the abdominal wall from the insertion of the trocars.

According to some studies, visceral pain accounts for most of the pain in the early postoperative period. Others state that the largest component in the total abdominal pain is from the incision sites, followed by the pneumoperitoneum and then the cholecystectomy [10]. This is in contrast to studies that show that infiltration of a local anesthetic agent at the trocar insertion sites does not provide pain relief, suggesting that the parietal pain does not contribute substantially to the total pain [11, 12]. In our study we evaluated the effect on the total pain score of the patient at different times, not the type or difference in intensities of the different types of pain. In our study, to minimize discrepancies, bupivacaine was injected at the trocar insertion sites as a routine procedure in both the control and case cohorts.

The cause of the shoulder pain has not been determined—proposed causes include neuropraxia of the phrenic nerve, stretching of the subdiaphragmatic fibers because of the pneumoperitoneum, increased stretch on the diaphragmatic attachments of the liver due to loss of visceral surface tension and peritoneal damage from chemical, ischemic or traumatic injury [13, 14]. Shoulder tip pain has been reported frequently ranging from 35 % to 63 %, but the intensity is markedly lower after laparoscopic cholecystectomy as compared to gynecologic procedures [15]. While visceral and parietal pain reduce after 24–48 h, shoulder pain may become more significant [16].

There is a dearth of local studies on the above subject. This study aims to provide impetus for further research and help in performance of laparoscopic cholecystectomy as a day care procedure.

### Patient and methods

This prospective observational control study was approved by the Liaquat national hospital and medical college ethical review board (Approval no #07895). Required sample size of 120 patients was calculated with 95 % confidence level and 90 % power. These patients were included consecutively in the study from June 2014 to December 2014 were recruited from the outpatient clinic after obtaining written and informed consent.

Inclusion criteria was only those patients who were undergoing laparoscopic cholecystectomy and also classified as American society of anesthesiology(ASA) I and II. Patients having acute cholecystits, laparoscopic converted to open surgery, surgery requiring placement of drains and patients with contraindication to Intravenous ketorolac use were excluded from this study.

All participants undergoing elective laparoscopic cholecystectomy were divided into two groups with concealment of the sequence and each group having equal number of patients. In the control group, 20 cc of normal saline solution without bupivacaine was irrigated at the surgical bed after laparoscopic cholecystectomy. The experimental group was irrigated with 20 cc of bupivacaine 0.5 % in normal saline solution. Participants were kept unaware of the treatment they received following the protocol of single blinded study.

Following the anaesthetic assessment, patients were admitted to the hospital the day before the operation. All patients received the same anaesthetic technique. Usual monitoring was used including heart rate, respiratory rate, continuous ECG, SpO2 and non-invasive arterial blood pressure. Creation of CO2 pneumoperitoneum at 14 mm Hg pressure was justified in all patients and standard laparoscopic cholecystectomy using the 4-port technique was performed. All the operations were performed by one team of surgeons that is experienced in laparoscopic surgery.

#### Prior hypothesis

Application of 0.5 % bupivacaine soaked surgicel at the gall bladder bed after laparoscopic cholecystectomy will result in a lower postoperative mean pain score compared to normal saline soaked surgicel.

#### Data collection

A performa was filled by the researcher for each patient, meeting inclusion criteria, in the ward, after taking written and informed consent and explaining him or her, the purpose of this study. The surgery was performed by a consultant having 5 years’ experience in laparoscopic cholecystectomy. Patients were allocated into two groups equally by balloting by the researcher, i.e. saline group and **bupivacaine** group. Participants were kept unaware of the treatment they received following the protocol of single blinded study. Both groups received 2 mg/kg of 0.5 % bupivacaine infiltrated at the port sites after the procedure to minimize the local pain. The members of bupivacaine group also had a 3 x 3 inch strip of surgicel soaked in 0.5 % bupivacaine placed at the gall bladder bed, and members of saline group had a surgicel soaked in normal saline placed at the gall bladder bed. Both groups received similar postoperative analgesia, I/V Ketorolac 30 mg at 8 h intervals during the study period. The assigned researcher used the VAS to measure pain scores at 4, 12 and 24 h after the procedure. All data, including age, sex, comorbid, duration of symptoms, duration of procedure, and VAS score at 4, 12 and 24 h after procedure, and the total pain score was recorded on a performa. The study was completed at the end of 24 h with no dropouts.

#### Statistical analysis

Data was analyzed by using SPSS version-19. Descriptive statistics, frequency and percentage were computed for qualitative variables like gender and pain control. Mean ± SD was computed for presentation of age and hospital stay. Chi square test was used to compare proportion difference between groups for gender and the independent sample *t* test of the significance was used to compare mean pain score between groups for continuous variables like age and pain score. *P* value ≤0.05 was taken as significant. Confounding variables like age, gender were controlled by stratification to see the effect on outcome (mean pain score).

## Results

A total of 120 patients undergoing laparoscopic cholecystectomy were included in this study. Patients were equally allocated into two groups according to treatment plan. The demographic characteristic of the two groups has shown that studied patients were matched as regarding gender, age, weight, ASA status and duration of surgery. However, most of studied patients were females (71.66 % and 80.3 % in saline and **bupivacaine** group respectively). Furthermore, mean operating time of surgery was not significantly different among the two groups (45.8, and 46.0 min in saline and **bupivacaine** group respectively). In addition, no significant differences were observed among two groups with respect to age, ASA status and weight (Table 1).

Post-operative abdominal pain was significantly lower in **bupivacaine** Group than Saline group. This difference was reported from 4 h till 12 h post-operatively. Although **bupivacaine** Group also reported lower pain scores after 24 h when compared with the saline group, however it is not statistically significant as reported (Table 2). Additional I/V analgesia was only required in one patient.

**Table 1:** Demographic characteristics of saline and bupivacaine group

	Saline Group	Bupivacaine Group	*P*-value
Male	17(28.33 %)	12(20.0 %)	0.394
Female	43(71.66 %)	48(80.0 %)	
ASA I	43(71.66 %)	17(28.33 %)	0.528
ASA II	47(78.33 %)	13(21.66 %)	
Age	43.0(11.86)	39.2(10.99)	0.073
Weight	81.28(4.15)	81.15(4.54)	0.867
Duration of Surgery	45.8(2.22)	46.0(2.11)	0.615

**Table 2:** Comparison of mean pain score between groups

	Saline Group	Bupivacaine Group	*P*-value
4 h	6.8(0.72)	6.2(0.89)	0.0005
12 h	4.3(0.97)	3.9(0.99)	0.022
24 h	2.6(0.80)	2.5(0.87)	0.57

**Table 3:** Perioperative characteristics of normal saline and bupivacaine group

	Saline Group	Bupivacaine Group	*P*-value
Shoulder tip pain	2(3.3 %)	1(1.7 %)	0.559
Bradycardia	6(10.0 %)	3(5.0 %)	0.491
Hypotension	610.0 %)	4(6.7 %)	0.743
Urinary retention	5(8.3 %)	2(3.3 %)	0.243
Pruritis	2(3.3 %)	1(1.7 %)	0.559
Ponv	2(3.3 %)	1(1.7 %)	0.559
Headache	1(1.7 %)	0(0 %)	0.315
Respiratory	1(1.7 %)	0(0 %)	0.315

**Table 4:** Postoperative outcomes in normal saline and bupivacaine group

	Saline Group	Bupivacaine Group	*P*-value
Time of oral intake (hr)	13.21(1.74)	11.78(1.93)	0.00
Time to walk (hr)	12.81(1.46)	12.20(1.83)	0.04
Length of Hospital stay	28.33(3.14)	26.31(2.07)	0.00

Bradycardia, Hypotension and Urinary retention were the most common perioperative symptoms reported, with an incidence of 28.3 % in the saline Group and 15 % in the **bupivacaine** group. There was no significant statistical difference. Three patients experienced shoulder tip pain, two from saline group and one from **bupivacaine** group with no significant difference. Six patients reported Prutitis and Ponv, four from saline group and two from **bupivacaine** group. Two patients reported headache and respiratory problems both of them were from saline group (Table 3).

Evaluation of postoperative details such as oral intake, time to walk and length of hospital stay revealed that bupivacaine group reported better outcomes as compared to saline group (Table 4).

## Discussion

Instillation of a local anesthetic agent in the subdiaphragmatic region as a method for pain control has been evaluated in many trials [17, 18]. While some series reported a benefit in terms of reduce postoperative pain, others showed no benefit [19–21]. Important factors that may be responsible for this difference in observations include the timing of the administration of the drug (before dissection versus after the procedure), volume of the solution used, and the method of drug instillation [22, 23]. Some authors believe that failure of adequate pain relief may be attributed to a short contact time of the drug with the surgical site due to the intraperitoneal influx [24, 25]. Using a sheet of oxidized cellulose (surgicel/tabotamp) soaked in bupivacaine, at the gall bladder bed is thought to increase the contact time of the drug, leading to better pain relief [26]. In our study we used a 2 inch × 3 inch strip of surgicel soaked in either bupivacaine or normal saline for the case and control groups respectively.

In our study, a total of 120 patients that fulfilled the inclusion criteria were included, after taking informed consent. All the patients were prescribed a standard postoperative analgesic regimen, which included intravenous ketorolac, 30 mg every 8 h.

The mean pain scores as calculated from the VAS were significantly less for **bupivacaine** Group, compared to saline Group at 4 h (*p* = 0.0005) and at 12 h (0.022), while the difference at 24 h was not significant (*p* = 0.57). Verma et al. noted a mean difference in the VAS of more than 10 between the case and control group, but this difference was not significant at 4 or 8 h after surgery [10]. This could be due to a small sample size with a large variation in the VAS at 4 h. Tariq et al. report a significant difference in the case group using bupivacaine plus ketorolac intraperitoneally, compared to bupivacaine alone [26]. Khan and coworkers also report bupivacaine at the gall bladder bed and infiltration at the port site to be an effective way to control postoperative pain [27]. Only one patient required additional analgesia in our study from the control group. Tariq et al also report that the difference in analgesia requirement was not significant [26].

Stratification according to gender revealed a significant difference in mean VAS in females at 4 and 12 h in females. Ure and coworkers have reported that females report more pain compared to males, therefore a significant difference in mean pain scores in females would further ascertain our hypothesis [4]. Data stratified according to age revealed a p value of 0.009, 0.14 and 0.67 at 4, 12 and 24 h for the less than 40 year age group, while for more than 40 years, these values were 0.003, 0.091, 0.174 respectively.

## Conclusion

In conclusion, there are considerable differences in the results from various studies. In our opinion, placing a surgicel soaked with bupivacaine at the gall bladder bed and infiltrating bupivacaine at the trocar insertion sites is a safe method, and decreases the postoperative pain resulting from laparoscopic cholecystectomy. We recommend that this method of analgesia be used routinely in surgery for symptomatic gall stones. Further research needs to be done to assess the efficacy and possible associated morbidity of this method in cases of acute cholecystitis.

### Consent

Written informed consent was obtained from the patients for publication of this article. A copy of the written consent is available for review by the Editor-in-Chief of this journal. Approval obtained from Liaquat national hospital and medical college ethical review committee.

## Abbreviations

I/V: Intravenous
VAS: Visual analogue scale
ASA: American society of anesthesiology

## References

[1] Bisgaard T, Klarskov B, Rosenberg J, Kehlet H (2001). Characterisics and prediction of early pain after laparoscopic cholecystectomy. Pain.

[2] Jensen K, Khlet H, Lund CM (2007). Post-operative recovery profile after laparoscopic cholecystectomy: a prospective, observational study of a multimodal anaesthetic regime. Acta Anaesthesiol Scand.

[3] Ure BM, Troidl H, Spangenberger W, Dietrich E, Lefering R, Neugebauer E (1994). Pain after laparoscopic cholecystectomy. Intensity and localization of pain and analysis of predictors in preoperative symptoms and intraoperative events. Surg Endosc.

[4] Michaloliakou C, Chung F, Sharma S (1996). Preoperative multimodal analgesia facilitates recovery after ambulatory laprocopic cholecystectomy. Anesth Analg.

[5] Barczynski M, Herman RM (2003). A prospective randomized trial on comparison of low-pressure and standard pressure pneumoperitoneum for laparoscopic cholecystectomy. Surg Endosc.

[6] Bisgaard T, Klarskov B, Kristiansen VB, Callesen T, Schulze S, Kehlet H, Rosenberg J (1999). Multiegional local anesthetic infiltration during laparoscopic cholecystectomy in patients receiving prophylactic multimodal analgesia: a randomized, double-blind placebo-controlled study. Anesth Analg.

[7] Wills VL, Hunt DR (2000). Pain after laparoscopic cholecystectomy. Br J Surg.

[8] Joris J, Thiry E, Paris P, Weerts J, Lamy M (1995). Pain after laparoscopic cholecystectomy: characteristics and effect of intraperitoneal bupivacaine. Anesth Analg.

[9] Mouton WG, Bessel JR, Otten KT, Madden GJ (1999). Pain after laparoscopy. Surg Endosc.

[10] Verma GR, Lyngdoh TS, Kaman L, Bala I (2006). Placement of 0.5 % Bupivacaine soaked Srgicel in the gall bladder bed is effective for pain after laparoscopic cholecystectomy. Surg Endosc.

[11] Adams WJ, Avramovic J, Barraclough BH (1991). Wound infiltration with 0.25 % buivacaine is not effective for postoperative analgesia after cholecystectomy. Aust N Z J Surg.

[12] Timoyiannis EC, Glantzounis G, Lakkas ET, Siakas P, Jabarin M, Tzourou H (1998). Intraperitoneal normal saline and Bupivacaine infusion for reduction of postoperative pain after laparoscopic cholecystectomy. Surg Laparosc Endosc.

[13] Woolf CJ, Mannion RJ (1999). Neuropathic pain: etiology, symptoms, mechanisms and management. Lancet.

[14] Dobbs FF, Kumar V, Alexander JI, Hull MG (1987). Pain after laparoscopic cholecystectomy related to posture and ring versus clip sterilization. Br J Obstet Gynaecol.

[15] Mravoic B, Jurisic T, Kogler-Majeric V, Sustic A (1997). Intraperitoneal bupivacaine for analgesia after laparoscopic cholecystectomy. Acta Anaesthesiol Scand.

[16] Elfberg BA, Sjovall-Mjoberg S (2000). Intraperitoneal bupivacaine does not effectively reduce pain after laparoscopic cholecystectomy: a randomized, placebo-controlled and double blind study. Surg Laprosc Endosc.

[17] Cunniffe MG, McAnena OJ, Dar MA, Calleary J, Flynn N (1998). A prospective randomized trial of intraoperative bupivacaine irrigation for management of shoulder tip pain following laparoscopy. Am J Surg.

[18] Lepner U, Goroshima J, Smarutel J (2003). Postoperative pain relief after laparoscopic cholecystectomy: a randomized double-blind clinical trial. Scan J Surg.

[19] Sarac AM, Aktan AO, Baykan N, Yagen C, Yalin R (1996). The effect and timing of local anesthesia in laparoscopic cholecystectomy. Surg Lap Endosc.

[20] Rademaker BM, Kalkman CJ, Odoom JA, Wit LD, Ringers J (1994). Intraperitoneal local anesthetics after laparoscopic cholecystectomy: effect on postoperative pain, metabolic responses and lung function. Br J Anesth.

[21] Pascqualucci A, De Angelis V, Contardo R, Colo F, Terrosu G, Donini A, Pasetto A, Bresadola F (1996). Preemptive analgesia: A randomized, double-blind, placebo-controlled study. Anesthesiology.

[22] Maestroni U, Sarli D, Devito C (2002). Pour Morard Kohan Brunaldi F, Anania G, Pavanelli L, Pasqualucci A, Donini A. A new method of preemptive analgesia in laparoscopic cholecystectomy. Surg Endosc.

[23] Schulte-Steinberg H, Weninger E, Jokisch D, Hofstetter B, Misera A, Lange V (1995). Intraperitoneal versus interpleural morphine or bupivacaine for pain after laparoscopic cholecystectomy. Anesthsiology.

[24] Scheinin B, Kellokumpu I, Lindgren L, Haglund C, Rosenberg PH (1995). Effect of Intraperitoneal bupivacaine on pain after laparoscopic cholecystectomy. Acta Anesthesiol Scand.

[25] Feroci F, Kroning KC, Scatizzi M (2009). Effectiveness for pain after laparoscopic cholecystectomy of 0.5 % Bupivacaine-soaked Tabotamp placed in the gall bladder bed: a prospective, randomized clinical trial. Surg Endosc.

[26] Tariq GR, Mian MA, Chaudry IA (2004). Comparison of intraperitoneal analgesia with Bupivacaine/Bupivacaine with Ketorolac after laparoscopic cholecystectomy. J Surg Pak.

[27] Khan SA, Butt K, Chaudry ZA, Mushtaq A (2000). Experience with 0.5 % Bupivacaine pro-peritoneal bleb after laparoscopic cholecystectomy- a new trend in analgesia. Ann King Edward Med Coll.

